# Supplementary material for: A systematic approach to the development of a safe live attenuated Zika vaccine
Source: Nat Commun. 2018 Mar 12;9:1031. doi: 10.1038/s41467-018-03337-2 (PMC5847552; doi:10.1038/s41467-018-03337-2)
Supplement: Supplementary file 1 — Supplementary Information [file 41467_2018_3337_MOESM1_ESM.pdf]

A systematic approach to the development of a safe live attenuated Zika vaccine

Kwek et al.

Supplementary Information

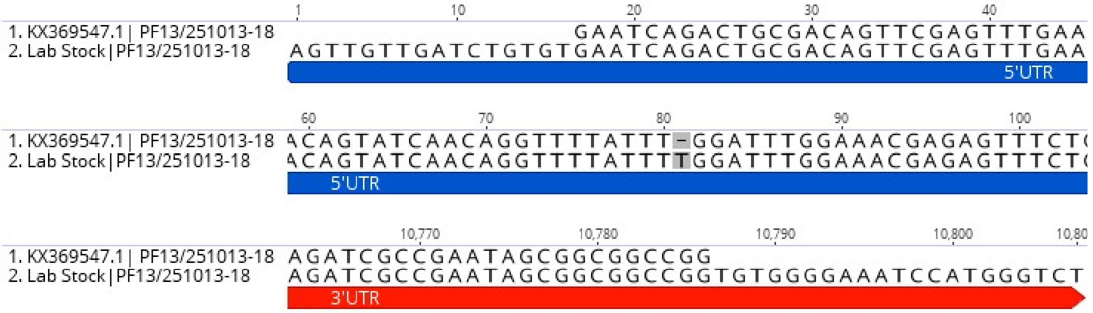

Supplementary Figure 1. Sequence differences between PF13 stock and published sequence. The full-length genomic sequence of PF13 (submitted under GenBank MG827392) was obtained through next-generation sequencing and mapped against the published sequence (GenBank KX369547) using the Geneious software. Differences in the non-coding regions of the 2 genomes are as shown.

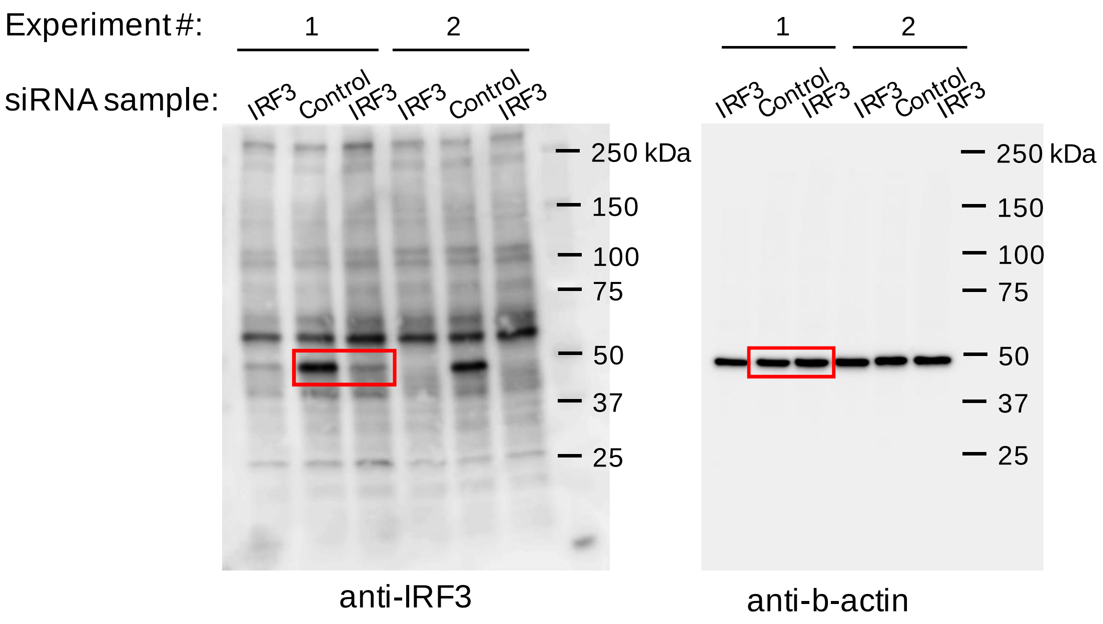

Supplementary Figure 2. Full blots of BHK-21 cell lysates after siRNA knockdown of IRF3. Western blots for IRF3 and β-actin on cell lysates from 2 independents experiments collected 48 hours after transfection of a control siRNA or siRNA for IRF3. Bands for IRF3 and β-actin presented in the main figure are indicated in red boxes. Molecular sizes as indicated.

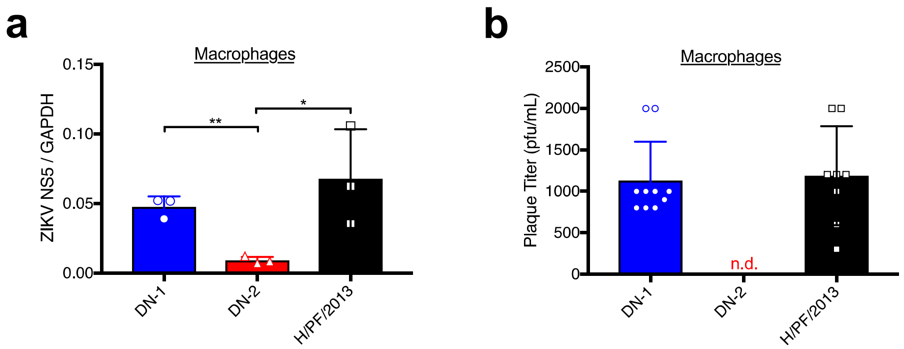

Supplementary Figure 3. Infectivity of DN-2 on monocyte-derived macrophages. Infectivity of DN-1, DN-2 and H/PF/2013 ZIKV strains on monocyte-derived macrophages (n=3) at 24 hours post-infection as determined (**a**) qPCR on infected cells and (**b**) plaque assay. Error bars represent s.d. *p<0.05, **p<0.01 in unpaired t-test. n.d. = not detectable.

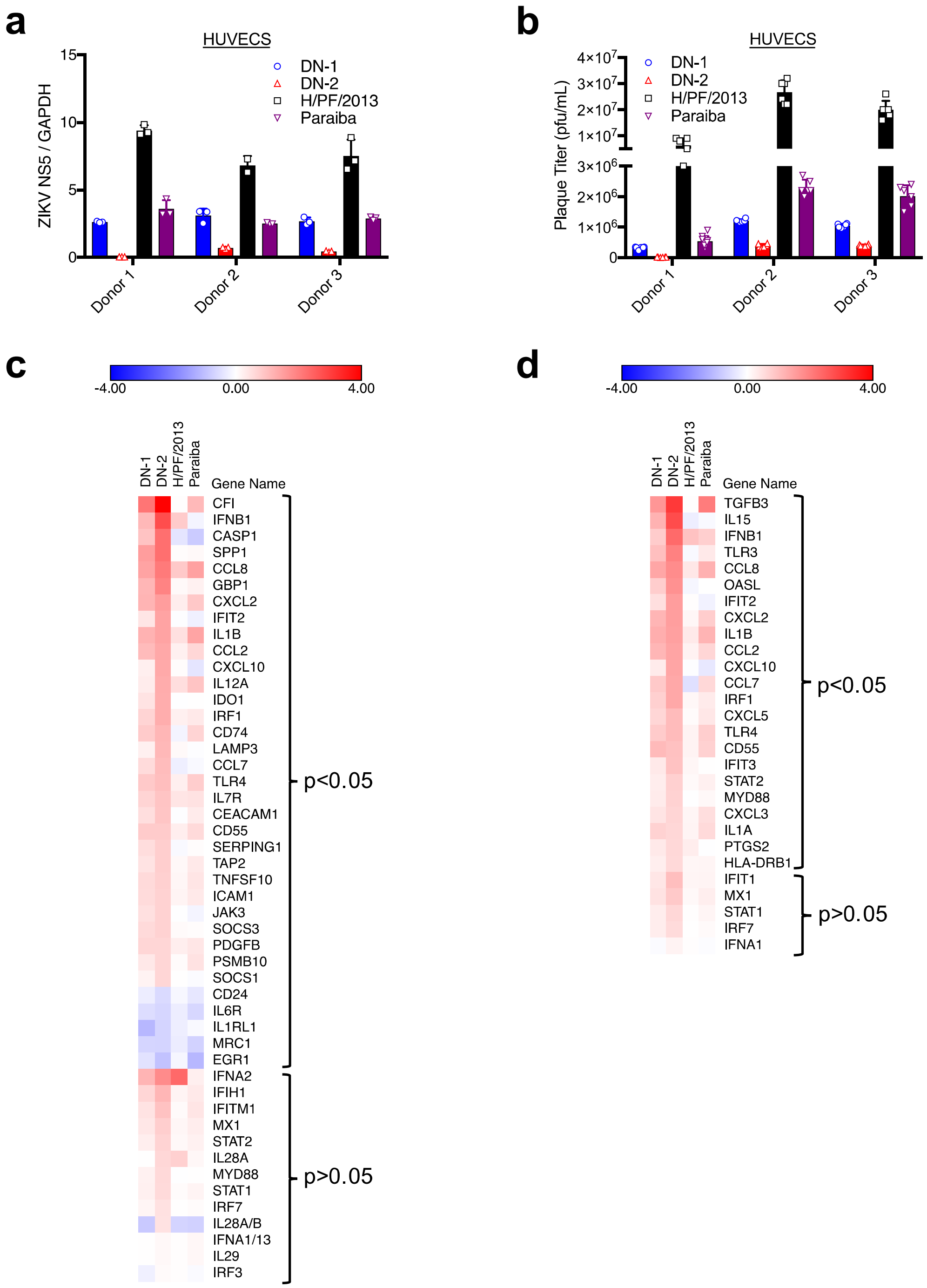

Supplementary Figure 4. Infectivity of DN-2 on HUVECs and immune responses in infected hESC-derived EPCs. Infectivity of DN-1, DN-2, H/PF/2013 and Paraiba ZIKV strains on HUVECs from 3 donors (n=3 per donor) were determined at 24 hours post-infection using (**a**) qPCR and (**b**) plaque assay. NanoString analysis on infected hESC-derived EPCs (n=3) were

performed using the (**c**) Immunology and (**d**) Inflammation pre-built panels by normalizing counts to in-built house-keeping genes within each sample and then normalized to uninfected samples (n=3). Heat maps represent $\log_2$ fold changes with $p < 0.05$ and $p > 0.05$ (Student's t-test in nSolver Analysis Software) for genes as indicated. NanoString data are from the same experiment shown in Figure 5(c) of main text. Error bars represent s.d.

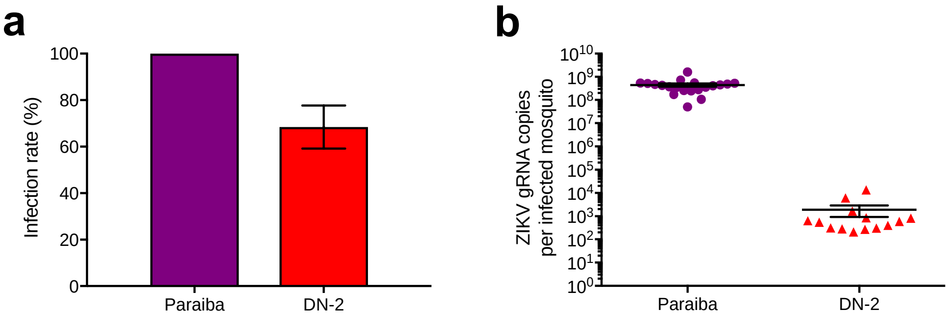

Supplementary Figure 5. Infectivity of DN-2 in *Aedes aegypti* mosquitoes. Mosquitoes were infected with the highly infective and transmissible Paraiba wild-type strain and DN-2 (n=20 per group). (**a**) Percent of mosquitoes with positive viral RNA detection in whole mosquitoes. (**b**) Viral RNA detected in the mosquitoes was quantified by qPCR. Error bars represent s.e.m.

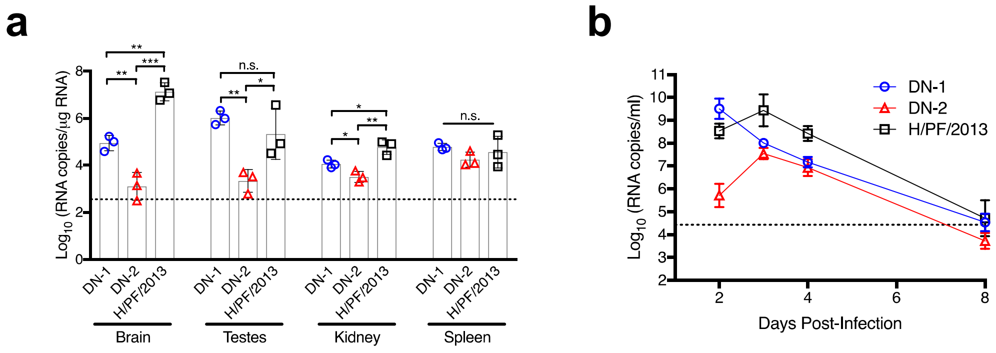

Supplementary Figure 6. DN-2 causes decreased organ infection and viremia during early infection. (**a**) Viral load in brains, testes, kidneys and spleens harvested at 8 days post-infection of male A129 mice (n=3) with 10^4 pfu of DN-1, DN-2 or H/PF/2013 were determined using qPCR. (**b**) Viremia levels in these mice at days 2, 3, 4 and 8 post-infection were measured by qPCR. Results shown are from same experiment performed for Figure 8(d) in main text. Error bars represent s.d. *p<0.05, **p<0.01, ***p<0.001 in unpaired t-test. Dotted lines on viral quantification graphs represent limit of detection.

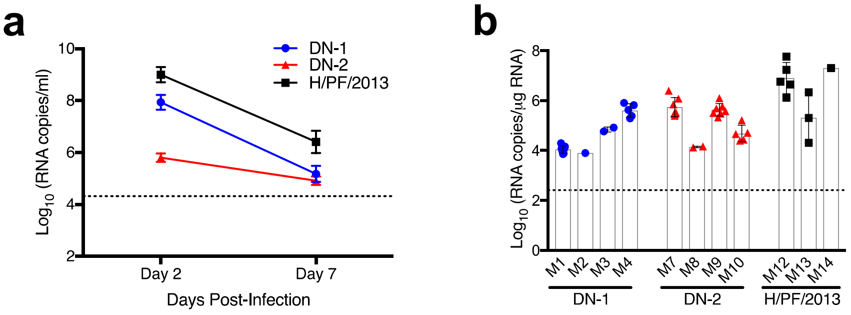

Supplementary Figure 7. Maternal viremia and placental viral loads in infected pregnant A129 mice. (**a**) Viremia in infected A129 dams (n=5 for DN-1 and DN-2, n=4 for H/PF/2013) infected with 10^3 pfu of each virus i.v. (**b**) Viral load in the placenta of infected pregnant mice was quantified using qPCR. Results shown are from same experiment performed for Figure 9 in main text. Error bars represent s.d.

Supplementary Table 1. Primers used in viral genome amplification for infectious clone generation.

**Primer Name**	**Primer Sequence (5'→ 3')**
**Fragment 1 (2137bp)**	
PF13 F1 Fwd	AGAGCTCGTTTAGTGAACCGAGTTGTTGATCTG
PF13 F1 Rev	GTGGATCAAGTTCCAGCATCATCTTAGAGTTCTCAGTGC
**Fragment 2 (2316bp)**	
PF13 F2 Fwd	GCACTGAGAACTCTAAGATGATGCTGGAACTTGATCCAC
PF13 F2 Rev	CACCTGCTCTTTCAATGTACATGTCCACACTCTTTCCTGA
**Fragment 3 (1832bp)**	
PF13 F3 Fwd	TCAGGAAAGAGTGTGGACATGTACATTGAAAGAGCAGGTG
PF13 F3 Rev	CTAAGCTTGAACTCTCCCTCAATGGCTGCTACTTTGTCG
**Fragment 4 (2570bp)**	
PF13 F4 Fwd	CGACAAAGTAGCAGCCATTGAGGGAGAGTTCAAGCTTAG
PF13 F4 Rev	CATACGGTGTGGTGTCGGTCATGGCTATTCCTGTGACTCC
**Fragment 5 (2145bp)**	
PF13 F5 Fwd	GGAGTCACAGGAATAGCCATGACCGACACCACACCGTATG
PF13 F5 Rev	ATGCCATGCCGACCCAGACCCATGGATTTCCCCACACCGG
**Vector Amplification**	
PF13 Vector Fwd	TGGGGAAATCCATGGGTCTGGGTCGGCATGGCATCTCCAC
PF13 Vector Rev	CACAGATCAACAACTCGGTTCACTAAACGAGCTCTGCT
